# Analysis of Clinical Samples of Pancreatic Cyst's Lesions with A Multi‐Analyte Bioelectronic Simot Array Benchmarked Against Ultrasensitive Chemiluminescent Immunoassay

**DOI:** 10.1002/advs.202308141

**Published:** 2024-01-17

**Authors:** Cecilia Scandurra, Kim Björkström, Mariapia Caputo, Lucia Sarcina, Enrico Genco, Francesco Modena, Fabrizio Antonio Viola, Celestino Brunetti, Zsolt M. Kovács‐Vajna, Cinzia Di Franco, Lena Haeberle, Piero Larizza, Maria Teresa Mancini, Ronald Österbacka, William Reeves, Gaetano Scamarcio, May Wheeler, Mario Caironi, Eugenio Cantatore, Fabrizio Torricelli, Irene Esposito, Eleonora Macchia, Luisa Torsi

**Affiliations:** ^1^ Dipartimento di Chimica and Centre for Colloid and Surface Science Università degli Studi di Bari Aldo Moro Bari 20125 Italy; ^2^ The Faculty of Science and Engineering Åbo Akademi University Turku 20500 Finland; ^3^ Dipartimento di Farmacia‐Scienze del Farmaco Università degli Studi di Bari “Aldo Moro” Bari 70125 Italy; ^4^ Department of Electrical Engineering Eindhoven University of Technology Eindhoven 5600 MB The Netherlands; ^5^ Center for Nano Science and Technology Istituto Italiano di Tecnologia Via Rubattino 81 Milan 20134 Italy; ^6^ Masmec Biomed – Masmec SpA division Modugno (BA) 70026 Italy; ^7^ Dipartimento Ingegneria dell'Informazione Università degli Studi di Brescia Brescia 25123 Italy; ^8^ CNR IFN Bari 70126 Italy; ^9^ Institute of Pathology Heinrich‐Heine University and University Hospital of Düsseldorf 40225 Duesseldorf Germany; ^10^ FlexEnable Technology Ltd Cambridge CB4 0FX UK; ^11^ Dipartimento Interateneo di Fisica Università degli Studi di Bari Aldo Moro Bari 70125 Italy; ^12^ Present address: Dipartimento di Ingegneria Elettrica ed Elettronica Università degli Studi di Cagliari Via Marengo 3 Cagliari 09123 Italy

**Keywords:** bioelectronic transistors, liquid biopsy, multivariate data processing, SIMOA‐single‐molecule‐array‐, SiMoT‐single‐molecule‐with‐a‐large‐transistor, single‐molecule biosensors

## Abstract

Pancreatic cancer, ranking as the third factor in cancer‐related deaths, necessitates enhanced diagnostic measures through early detection. In response, SiMoT‐Single‐molecule with a large Transistor multiplexing array, achieving a Technology Readiness Level of 5, is proposed for a timely identification of pancreatic cancer precursor cysts and is benchmarked against the commercially available chemiluminescent immunoassay SIMOA (Single molecule array) SP‐X System. A cohort of 39 samples, comprising 33 cyst fluids and 6 blood plasma specimens, undergoes detailed examination with both technologies. The SiMoT array targets oncoproteins MUC1 and CD55, and oncogene *KRAS*, while the SIMOA SP‐X planar technology exclusively focuses on MUC1 and CD55. Employing Principal Component Analysis (PCA) for multivariate data processing, the SiMoT array demonstrates effective discrimination of malignant/pre‐invasive high‐grade or potentially malignant low‐grade pancreatic cysts from benign non‐mucinous cysts. Conversely, PCA analysis applied to SIMOA assay reveals less effective differentiation ability among the three cyst classes. Notably, SiMoT unique capability of concurrently analyzing protein and genetic markers with the threshold of one single molecule in 0.1 mL positions it as a comprehensive and reliable diagnostic tool. The electronic response generated by the SiMoT array facilitates direct digital data communication, suggesting potential applications in the development of field‐deployable liquid biopsy.

## Graphical abstract

SiMoT‐Single‐Molecule with Large Transistor technology simultaneously analyzes protein and genetic markers, achieving a one‐molecule threshold in 0.1 mL. Benchmarking against SIMOA chemiluminescent ultrasensitive assay, SiMoT outperforms SIMOA in speed and overall performance. Moreover, SiMoT provides an electronic response, enhancing its suitability for direct digital data communication.

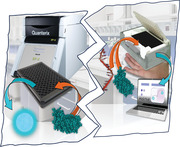

## 1 Introduction

Cancer is responsible for almost one out of six deaths annually globally.^[^ ^1^ ^]^ Furthermore, pancreatic ductal carcinoma ranks as the third cause of mortality correlated to cancer, with fewer than 9% of patients surviving five years following their diagnosis.^[^ ^2^ ^]^ The unfavorable prognosis for those patients is the result of 80–85% of cases being detected in advanced stages, where either the tumor has invaded major surrounding vessels or distant metastases have become apparent.^[^ ^3^ ^]^ Hence, it is evident that an improved prognosis is linked to the early detection of pancreatic lesions. Specifically, the ability to classify precursors such as the pancreatic lesions as either benign non‐mucinous or high‐ or low‐grade mucinous cysts, would prove highly advantageous in determining the optimal course of treatment and improving prognosis. From this standpoint, recognizing high‐grade cysts holds significant importance due to their increased susceptibility to progress into invasive cancer.^[^ ^4^ ^]^

Therefore, the identification of biomarkers in the bloodstream emerges as a valuable strategy for the timely and minimally invasive cancer diagnosis. The concept underlying this approach often referred to as “liquid biopsy”, is that cancer cells turn over frequently, releasing in the circulation mutated oncogenes and oncoproteins. To this aim, the diagnostic sensitivity of the analytical test may pose a substantial constraint on the successful implementation of liquid biopsies. Indeed, research studies indicate that individuals with early‐stage cancers may host down to one mutated gene or protein per 0.1 mL of plasma, necessitating detection limits in the 10 zeptomolar, zM, range (10^−20^ M).^[^ ^5^, ^6^ ^]^ Historically, mutated DNA genes, such as *KRAS* and *TP53*,^[^ ^7^, ^8^ ^]^ released by cancer cells have been explored as suitable pancreatic cancer precursors biomarkers, relying on the capability of Polymerase Chain Reaction (PCR)^[^ ^9^ ^]^ based assays and Next Generation Sequencing (NGS)^[^ ^10^ ^]^ to track oncogenes down to the single copy in 0.1 mL (10 zM concentration). However, plasma DNA‐based assays have demonstrated limited sensitivity for early‐stage cancers,^[^ ^5^ ^]^ while the combined assay of oncogenes and oncoproteins has the potential to enhance sensitivity in detecting pancreatic cancers.^[^ ^11^ ^]^ Therefore, a parterre of markers, comprising both proteins and genes, should be assayed to detect solid tumors at an early stage with diagnostic‐specificity and sensitivity ≥ 95%.^[^ ^7^ ^]^ The main hindrance to achieving this goal lies in the limited availability of immunometric platforms capable of effectively operating at extremely low concentrations of target biomarkers.^[^ ^6^ ^]^ The enzyme‐linked immunosorbent assay (ELISA) still stands as the workhorse for immunoassays, with a limit of detection ranging from the nanomolar (nM, 10^−9^ M) to picomolar (pM, 10^−12^ M) concentrations.^[^ ^12^ ^]^ Prominent developments within this domain encompass ELISA transition into the “digital” realm, resulting in the advent of Single‐Molecule Assay (SIMOA) technology by Quanterix.^[^ ^13^ ^]^ The SR‐X SIMOA platform enables multiplexing assays of proteins with limit‐of detections (LODs) below fM (reaching down to 220 zM, corresponding 10–10^5^ molecules in a volume of 0.1 mL).^[^ ^14^ ^]^ Commercially available are various Ready‐to‐use SIMOA kits and Homebrew SIMOA kits designed for customizable assays, facilitating the detection of protein markers in neurology, oncology, and immunology. It's worth mentioning that, while a SIMOA assay for DNA detection has been successfully developed in the laboratory as a proof‐of‐principle, obtaining a LOD of 0.07 fM,^[^ ^15^, ^16^ ^]^ it has yet to be commercialized. A swifter and more practical version of the SIMOA platform, referred to as SP‐X planar technology,^[^ ^17^ ^]^ has been recently introduced, albeit achieving LODs in the attomolar range (10^−18^ M) at best.^[^ ^18^ ^]^

The emergent SiMoT – Single Molecule with a large Transistor, developed at TRL‐Technology Readiness Level 5 as a single‐sensor and as a 96‐multiplexing array, has been proven capable of assay at a LOD of one single marker, both proteins and oligonucleotides.^[^ ^19^, ^20^, ^21^, ^22^, ^23^, ^24^, ^25^, ^26^, ^27^, ^28^, ^29^ ^]^ This pioneering technology utilizes an Electrolyte‐gated organic field‐effect transistor (EG‐OFET)^[^ ^30^ ^]^ linked to a disposable cartridge housing the gate electrode, which functions as a detecting interface. The sensing gate covers dimensions ranging from micro to millimeters (µm^2^–mm^2^ wide), accommodating 10^11^–10^12^ cm^−2^ biorecognition elements.^[^ ^31^ ^]^ Notably, SiMoT operates without the requirement for labels, as its electronic output signal is intricately linked to gate work function modification triggered by the interaction between the biomarker and its recognition element.^[^ ^32^, ^33^ ^]^ Due to the generality of the amplification mechanism underlying SiMoT expectational LODs,^[^ ^6^ ^]^ such technology provides the opportunity to target a plethora of different diseases. This is achieved by employing a suitable biorecognition element, such as the capturing antibody (for targeting a specific antigen/protein) or the probe (for binding a specific oligonucleotide), integrated into the sensing gate electrode. In recent studies, the SiMoT single sensor was employed in the analysis of a singular marker/pathogen, such as the *COVID‐19* virus and the *Pierce's disease* bacterium.^[^ ^26^, ^27^ ^]^ Meanwhile, a 96‐multiplexing array, developed in the framework of the H2020 EU project: “Single‐molecule bioelectronic smart system array for clinical testing – SiMBiT”,^[^ ^34^ ^]^ showcased its capability for concurrently analyzing various markers, namely oncoproteins Mucin 1 (MUC1) and Complement‐decay‐accelerating factor (CD55), and mutated oncogene *KRAS^G12D^*, in blood samples from patients with pancreatic cancer.^[^ ^35^ ^]^ The SiMoT array underwent a pre‐clinical trial, analysing cyst fluids and blood plasma from 47 patients. Impressively, the examination of all three markers demonstrated a false negative rate within 1–5%, with no false positive error. The output of the SiMoT array underwent benchmarking against histo/cytopathology, supplemented by the analysis of mutated oncogenes *KRAS*, *GNAS*, and TP53 using NGS. Notably, the SiMoT array demonstrated superior performance compared to state‐of‐the‐art diagnostic methods, relying solely on genomic marker analysis, particularly in terms of diagnostic sensitivity. The enhanced capabilities of the SiMoT array in comparison to established diagnostic procedures are likely related to its capacity to detect oncoproteins down to the physical limit.

In the present study, an extensive benchmarking analysis is reported to assess the performance level of the SiMoT array against the SIMOA SP‐X Imaging and Analysis System. A cohort of 39 samples, comprising 33 cyst fluids and 6 blood plasma specimens, underwent thorough examination utilizing both the SiMoT array – targeting oncoproteins MUC1 and CD55, and oncogene *KRAS –* and the SIMOA SP‐X planar technology, focused exclusively on MUC1 and CD55. The analysis of MUC1 and CD55 oncoproteins, alongside mutated oncogenes typically assessed via NGS, is proposed and benchmarked against the widely recognized digital ELISA workhorse here for the first time.

Multivariate data processing was applied to interpret the complex data patterns generated by these advanced technologies. Specifically, Principal Component Analysis (PCA), fed by data collected with the SiMoT array, revealed that its multiplexing capabilities effectively discriminate high‐grade, low‐grade, and non‐mucinous cysts, representing a notable advancement in cystic lesion characterization. Conversely, the PCA analysis applied to SIMOA assay data indicated rather non‐effective substantial differentiation among the three cyst classes. Beyond its diagnostic efficacy, the SiMoT technology emerges as a more cost‐effective and expeditious alternative to the SIMOA SP‐X planar assay. A distinctive feature is SiMoT unique ability to concurrently analyse protein and genetic markers with the threshold of one single‐molecule, establishing it as a singular and comprehensive diagnostic tool. Furthermore, the electronic response generated by the SiMoT array positions it favourably for direct digital data communication, suggesting potential applications in the development of field‐deployable liquid‐biopsy diagnostics.

## 2 Results and Discussion

### 2.1 SiMoT Multiplexing Array

**Figure 1:**
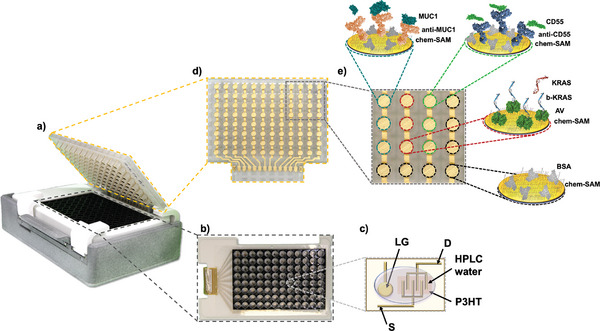
a) Image of the portable SiMoT ELISA‐like 96‐sensors bioelectronic array, developed within the SiMBiT project framework. b) Overhead perspective of the 96 Electrolyte‐Gated Organic field effect transistors (EGOFETs) defined on a PEN substrate with the bottomless ELISA plate glued on top. c) Overhead perspective of the 3D‐sensing gate used as SiMoT array lid. d) Enlarged view of a designated region for the assay of one patient's plasma or cyst's fluid sample, encompassing 16 sensing gates. The color indicators represent biofunctionalized gates tested in triplicate, incorporating specific antibodies targeting MUC1 (cyan) and CD55 (green), or probes designed for the binding of mutated KRAS (red). Furthermore, each patient's sample is subjected to 7 gates coated with BSA (black) for negative‐control experiments.

The SiMoT multiplexing array, comprises a 96‐wells ELISA‐like array of biofunctionalized sensing electrodes.^[^ ^35^ ^]^ The SiMoT array, as illustrated in **Figure** **1**a, consists of a disposable cartridge sharing the design specification of an 8×12 ELISA plate. Additionally, it is endowed with a reusable reader, comprising the PCB module and the Silicon‐Integrated Circuit (Si‐IC) that connects to a standard smart device via USB for operation. The fabrication specifics of the SiMoT array are extensively outlined elsewhere.^[^ ^35^ ^]^ To provide a brief overview, the disposable cartridge comprises an array of 96 Electrolyte‐Gated Organic field effect transistors (EGOFETs) fabricated on a plastic foil (Figure 1b). Those EGOFETs integrate a lateral gate (LG) designed to monitor device stability, lying in the same PEN foil. The device is equipped with interdigitated gold electrodes for the source (S) and drain (D), coated with a conjugated polymer, specifically poly(3‐hexylthiophene) (P3HT), via ink‐jet printing (Figure 1c). Enclosed within a bottomless ELISA plate, each EGOFET facilitates the dispensing of 0.3 mL of water (HPLC grade). This water plays a crucial role in connecting the gates to the channel through charge double layers. The disposable cartridge is equipped with OTFT multiplexing electronics and a 3D sensing gate plate (Figure 1d). The latter consists of 96 plastic pillars extending from a planar substrate fabricated through 3D printing stereolithography (SLA). This method allows for swift and flexible design without unnecessary material waste, delivering a cost‐effective solution with precision and compatibility across a broad spectrum of materials. The design of the 3D gate pillars considered two primary constraints, namely the geometries conforming to those of the ELISA plate, and the minimization of fluid usage for biofunctionalizations to mitigate fabrication costs. Gold deposition was performed through e‐beam evaporation, allowing to define the round detecting interface along with the pathways to the connectors. The deposited gold has an approximate thickness of 150 nm. Following this, the gates undergo biofunctionalization, where the biorecognition elements are covalently attached to the gold gate, ensuring a high level of selectivity in binding to a particular marker. As illustrated in Figure 1e and Figure 6 areas for each 3D sensing gate plate can be identified, each one comprising 16 pillars engaged in the assay of one single patient's body fluid. Each patient undergoes testing with three replicates for each of the three biomarkers, accompanied by seven negative control experiments, encompassing Bovine Serum Albumin (BSA) coated sensing gates exposed (black, Figure 1e) to the patient's body fluid. The capturing antibodies anti‐MUC1 (cyan, Figure 1e), and anti‐CD55 (green, Figure 1e) are covalently coupled to the 3D sensing gates’ gold surface. For the probe detecting *KRAS*, first avidin (AV) is covalently bounded to the gold gate surface. Then the affinity binding among AV and a biotinylated oligonucleotide strand (b‐*KRAS*), which hybridizes with *KRAS^mut^*, occurs (red, Figure 1e).

#### 2.1.1 Characterization and Optimization of the Biofunctionalized Sensing Gates

**Figure 2:**
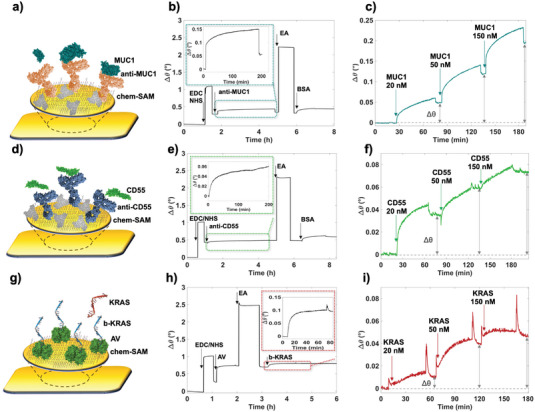
Pictorial representations and Surface Plasmon Resonance (SPR) characterizations of the biofunctionalization protocols involving a–c) anti‐MUC1 and d–f) anti‐CD55 capturing antibodies, while panel g–i) pertains to b‐KRAS probes. The assays for MUC1, CD55, and KRAS standard solutions were conducted, examining a concentration range from 20 nM to 150 nM. All sensograms are presented as the average of two replicates.

The biofunctionalization procedure of the 3D‐sensing gates plate was independently evaluated and optimized using Multi‐Parametric Surface Plasmon Resonance (MP‐SPR) in the Kretschmann configuration.^[^ ^36^ ^]^ The SPR system featured dual laser sources, both emitting light at a 670 nm wavelength, directed toward areas on the specimens spaced 3 mm apart, to assess for its uniformity. SPR characterization was engaged in the real‐time examination of the modified gold surface featuring biorecognition elements, specifically anti‐MUC1 and anti‐CD55 capturing antibodies, along with the genetic probe b‐*KRAS*. This method also yielded the respective surface coverage for each of these biorecognition elements.^[^ ^37^, ^38^, ^39^, ^40^ ^]^ To this aim, BK7 glass coated by a 40 nm gold film on a 2 nm chromium adhesion layer was used as the SPR sensor slide, after standard cleaning procedures.^[^ ^37^, ^38^ ^]^ The gold surface was left in contact overnight with a 10 mM thiol solution mixture of 11‐mercaptoundecanoic acid and 3‐mercaptopropionic acid (11‐MUA: 3‐MPA; 1:10 molar ratio). The modified slides, comprising the chemical self‐assembled monolayer (chem‐SAM) of thiols, were successively introduced into the SPR cell. The optical signal is recorded as the variation of the SPR resonance angle, *Δθ* (°), as a function of time, hereafter mentioned as “sensogram”. The subsequent steps of the biofunctionalization protocol were conducted in situ for the three distinct biorecognition elements, as depicted in **Figure** **2a,d,g**. This involved injecting each reagent into the 0.1 mL SPR flow cell and monitoring the sensogram in real time. Figure 2b,e,h show the sensograms acquired in the three biofunctionalization procedures. In all these cases, the chem‐SAM carboxylic terminal groups were activated by a 1‐Ethyl‐3‐(3‐dimethylamino‐propyl)carbodiimide (EDC) 0.2 M and N‐hydroxysulfosuccinimide sodium salt (sulfo‐NHS) 0.05 M solution in HPLC water for 20 min, and then rinsed with phosphate buffer saline solution PBS (ionic strength 162 mM, pH 7.4). Subsequently, the activated surface was exposed to either to the anti‐MUC1 (Figure 2b) or to the CD55 (Figure 2e) solutions (20 µg mL^−1^) in PBS, to be covalently attached to the SAM. After 3.5 h PBS was injected to rinse any antibody excess from the surface. The Ethanolamine hydrochloride (EA) 1 M solution in PBS was injected and left in contact for 45 min to saturate the chem‐SAM unreacted sites. Finally, a BSA solution (0.1 mg mL^−1^) in PBS was injected and left to interact for 1 hour with the surface, to address potential gaps in the biolayer and mitigate aspecific adsorption in the assay.

For the biofunctionalization protocol involving the immobilization of the b‐*KRAS*, the procedure shown in Figure 2h was used. The activated chem‐SAM was exposed to an AV solution (50 µg mL^−1^) in PBS for 1 h. Subsequent to a PBS rinse, the surface treated with AV‐modified SAM underwent an additional 45‐minute treatment with 1 M EA in PBS. Finally, the b‐*KRAS* solution (5 µg€mL^−1^) in PBS was injected and left in contact for 1 h.

The SPR sensograms shown in the insets of Figure 2b,e,h were used to calculate the surface mass‐density Г (ng cm^−2^) of the biorecognition elements deposited on the chem‐SAM.^[^ ^39^, ^41^ ^]^ The surface mass‐density can be evaluated by combining the Jung model^[^ ^41^ ^]^ and de Feijter's equation,^[^ ^42^ ^]^ assuming a linear approximation at small layer thicknesses.^[^ ^40^ ^]^ Therefore it is possible to establish a direct proportionality between Г and Δθ:

(1) $$\Gamma =\frac{\delta }{S}\cdot \Delta \theta \cdot {\left(dn/dC\right)}^{-1}$$

where δ represents the attenuation distance of the evanescent electric field with a specified value of 100 nm, S denotes the constant for bulk sensitivity measured in degrees per refractive index unit (86.3° at a wavelength of 670 nm), and dn/dC signifies the specific refractivity of the adsorbed biolayer of 0.182 cm^3^ g^−1^.^[^ ^43^ ^]^ Under these assumptions and by substitution of those values in Equation 1, the surface mass density Γ is evaluated by analyzing the observed angular shift in experimental data., as in Equation 2:

(2) $$\Gamma =\Delta \theta \cdot 637\;\left[ng/cm^{2}\right]$$

The surface mass density calculated according to Equation 2 for the three biofunctionalization procedures with anti‐MUC1, b‐KRAS and anti‐CD55 is reported in **Table** **1**.

**Table 1:** Surface coverage calculated for the three biofunctionalization procedures with anti‐MUC1, anti‐CD55, and b‐KRAS.

Biorecognition element	Δθ [°]	Г [ng·cm^−2^]	Г [molecules·cm^−2^]
anti‐MUC1	0.056 ± 0.002	36 ± 1	(1.24 ± 0.04)·10^11^
anti‐CD55	0.059 ± 0.002	38 ± 1	(1.31 ± 0.04)·10^11^
b‐KRAS	0.096 ± 0.001	61.2 ± 0.1	(4.01± 0.01)∙10^12^

In each instance, the surface density of biorecognition elements is ≈10^12^ molecules⋅cm^−2^, nearing the maximum achievable packing of biorecognition elements on a surface, which is 10^4^ µm^−2^.^[^ ^44^ ^]^ The SPR sensograms shown in the insets of Figure 2b,e,h also prove that an exposure time of 2 h is needed to obtain 95% of the surface mass density in the case of the anti‐MUC1 and anti‐CD55 depositions, while only 1 hour is sufficient in the case of b‐*KRAS*. Indeed, those exposure timeframes still lead to biorecognition elements’ surface densities as high as (1.17 ± 0.04)·10^11^ anti‐MUC1·cm^−2^, (1.23 ± 0.04)·10^11^ anti‐CD55·cm^−2^, and (4.01 ± 0.01)∙10^12^ b‐KRAS cm**^−^** ^2^.

The modified SPR slides were subsequently exposed to the corresponding ligands to validate the effectiveness of the immobilized elements in recognizing the target biomarkers (Figure 2c,f,i). For this purpose, PBS buffer served as a reference fluid for establishing the baseline. Following this, successive injections of 0.1 mL MUC1, CD55, *KRAS* standard solutions were carried out at increasing concentrations, covering a concentration range from 20 nM to 150 nM. The binding of MUC1 at the maximum inspected concentration of 150 nM (Figure 2c) resulted in *Δθ* = (0.190 ± 0.002)°, equivalent to a surface density of (1.84 ± 0.2)·10^12^ molecules·cm^−2^, while for CD55 proteins (Figure 2f) an angle variation of *Δθ* = (0.073 ± 0.002)° was registered, corresponding to a surface density of (6.6 ± 0.2)·10^11^ molecules·cm^−2^. Moreover, the binding of *KRAS* at a concentration of 150 nM (Figure 2i) produced a Δθ = (0.046 ± 0.002)°, corresponding to a surface coverage of (1.95 ± 0.2)·10^12^ molecules·cm^−2^. The surface densities of these ligands conform to a dense deposition of biomarkers on the capturing layer, and they align closely with the amount of binding sites accessible on the SPR slide. The observed SPR angle shift with MUC1, CD55, and *KRAS* is akin to the Δθ attained in previously published SPR assays,^[^ ^45^ ^]^ validating the capturing efficacy of the biofunctionalization protocol devised for the SiMoT 3D‐sensing gate plate.

#### 2.1.2 SiMoT Multiplexing Assay of Oncoproteins and Oncogenes

**Figure 3:**
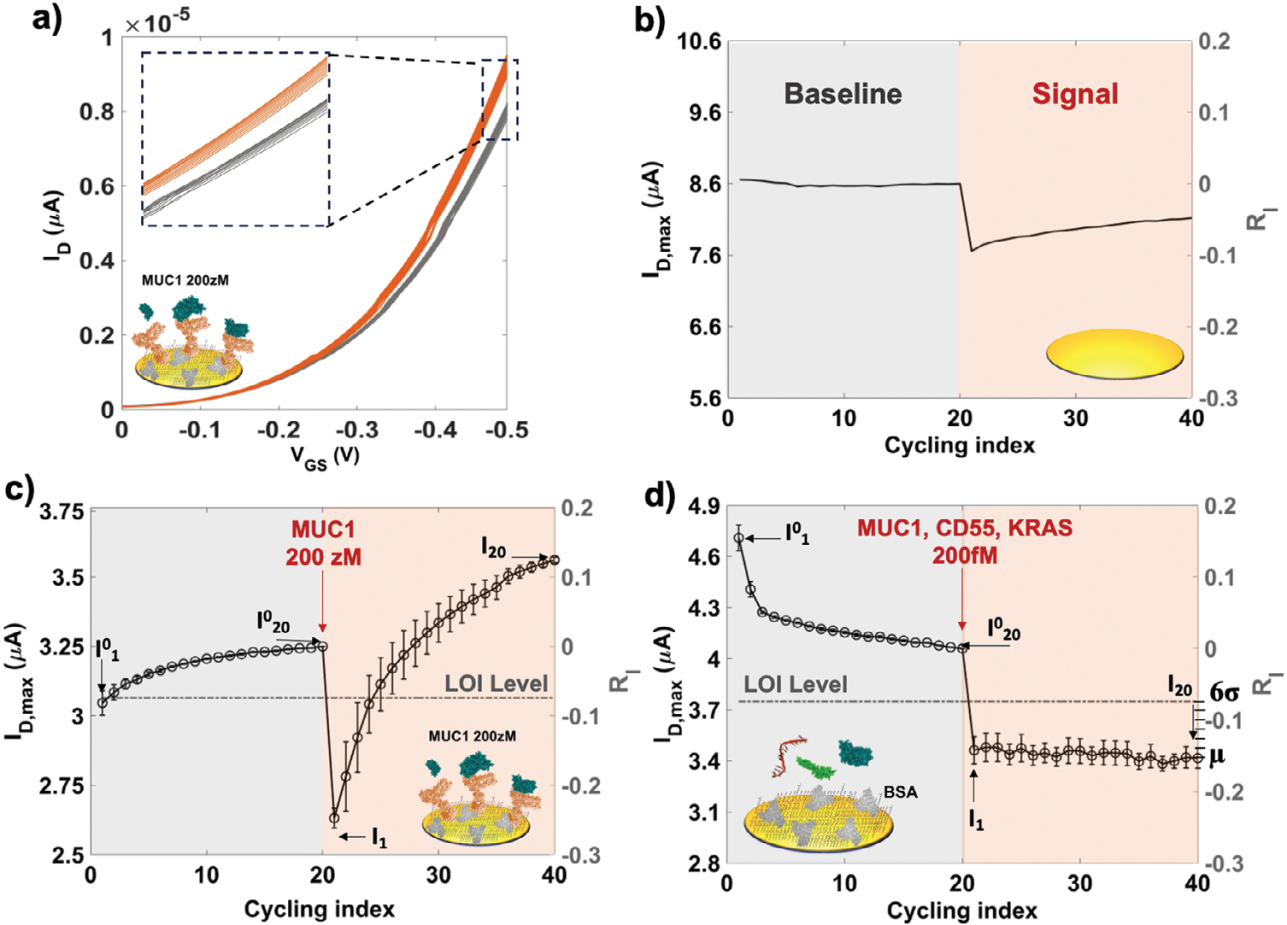
a) Typical sensing signal (red curves) and baseline signal (black curves) observed throughout the cycling process, involving 20 transfer characteristics (I_D_ versus V_GS_ in the 0 V to −0.5 V window at a stable V_D_ = −0.4 V). The baseline is recorded using a 3D sensing gate biofunctionalized with anti‐MUC1 following incubation in PBS reference fluid. The sensing signal is taken using the same 3D sensing gate following incubation in a PBS standard solution of MUC1, containing 12 ± 3 molecules in 0.1 mL. The relative current change R_I_ = (I – I^0^)/ I^0^ is plotted against the cycling index for b) the bare gold lateral reference gate, c) the anti‐MUC1 biofunctionalized 3D sensing gate, and d) the BSA‐coated 3D sensing gate used as a negative control experiment. R_I_ values are recorded at V_GS_ and V_D_ of −0.4 V, for each cycling index. Average values for data points were obtained from three replicates across three separate gates, and error bars were calculated as relative standard deviations. The data recorded after incubation in the reference fluid (PBS) is denoted by a black shadow, while measurements following incubation in a PBS standard solution of the target analyte are represented by a red shadow. The dashed‐dotted grey line represents the Limit‐of‐Identification (LOI) threshold.

The SiMoT multiplexing sensing protocol allows the examination of body fluids from six patients simultaneously utilizing a single 3D sensing gate cover plate, as outlined in the following. The sensing protocol involves an initial incubation step of the sensing gates for 10 min into an ELISA plate, where PBS reference fluid was dispensed in each well. Subsequently, the sensing plate undergoes thorough washing with HPLC grade water before being positioned onto the SiMoT 96‐wells ELISA‐like array. The SiMoT array is subsequently operated, recording a cycle of 20 consecutive transfer characteristics. Those are obtained by measuring the drain current (I_D_) varying with the gate potential (V_GS_), which is swept in the range from 0 V to −0.5 V, while maintaining the drain voltage at −0.4 V.^[^ ^46^, ^47^ ^]^ This procedure, hereafter named “cycling”, is implemented in each of the 96 EGOFETs, dealing with one column at a time. The acquisition of the cycling process necessitates 2.5 minutes per column of the EGOFET array. Consequently, the multiplexing electronics require 30 minutes to complete the cycling of the entire SiMoT array, addressing 12 columns of EGOFET. A typical set of 20 transfer curves, registered with a sensing gate coated with anti‐MUC1 after exposure to the reference fluid, is depicted in **Figure** **3**a as black lines. This cycling serves to define the *baseline I^0^*. Following that, the same 3D sensing gate plate is incubated for 10 min into an ELISA plate filled with 0.1 mL of the solutions to be assayed. Once the incubation step is completed, the 3D sensing gate plate undergoes washing and is then reinstated as the lid of the SiMoT array. Subsequently, a new cycle of transfer characteristics is acquired, defining the sensing signal *I*. A representative cycling of transfer characteristics, recorded with the anti‐MUC1 coated gate upon exposure to a PBS standard solution of MUC1 at a concentration of 200 zM is illustrated in Figure 3a as red curves. The change in the current signal becomes evident with just 12 ± 3 molecules present in the 0.1 mL sampled solution. Throughout each incubation step of the sensing gate cover plate, the array of EGOFETs undergoes cycling in water, sweeping the reference lateral gate (LG) coplanar to the source and drain electrode. As illustrated in Figure 3b, two cycling sets are recorded using the reference LG during the incubation periods of the 3D sensing gate plate in both the reference fluid and the assayed samples. Specifically, the relative current changes R_I_ = (I – I^0^)/I^0^, where *I* and *I^0^* are acquired at V_GS_ and V_D_ = −0.4 V, versus cycling index are presented in Figure 3b for the reference LG. Crucially, the R_I_ values recorded with the reference LG reach a maximum of 5%, demonstrating the high stability of the EGOFET array. In cases where a particular EGOFET exhibits a current shift exceeding 10%, the sensing signal obtained from that specific device is disregarded. The entirety of the sensing process, inclusive of assessing the stability of EGOFETs, to evaluate three markers across 5–6 patients necessitates a total duration of 90 min. In Figure 3c, the R_I_ shift is depicted following the incubation of an anti‐MUC1 biofunctionalized gate first in the reference fluid (grey shadowed) and then in a 200 zM standard solution of MUC1 (red shadowed). The curve represents the average of currents measured for three replicates, with the error bars indicating the data dispersion over one standard deviation (1σ).

In contrast, Figure 3d depicts the outcomes from negative‐control trials. In this scenario, the sensing gates pillars, coated with BSA, is incubated into PBS solutions encompassing 200 fM of the three markers together (MUC1, CD55, and *KRAS*), equivalent to 10^7^ molecules in 0.1 mL. The concentration mentioned is significantly greater, spanning six orders of magnitude beyond those utilized in the sensing experiment illustrated in Figure 3c. Despite these substantially elevated concentrations, it has been shown that the non‐capturing BSA element does not exhibit binding to any of the markers. Under these circumstances, the curve showed in Figure 3d represents the mean of the currents recorded across seven control experiments, while the error bars are the data dispersion over 1σ. The average value of the R_I_ registered with the negative control experiment μ, along with its relative standard deviation provides the Limit‐of‐Identification (LOI) level. Indeed, the SiMoT array is designed as a binary assay, with the decision threshold set at the LOI.^[^ ^35^ ^]^ The latter establishes the minimal quantity of the specific analyte that can be differentiated from the statistical fluctuations with level of confidence exceeding 99%, ensuring false‐positive and false‐negative rates below 1%.^[^ ^48^ ^]^ That is guaranteed by positioning the LOI at the noise‐average level, determined as the mean of the signal recorded during the negative control experiment (µ), increased by six times its standard deviation (6·σ),^[^ ^49^ ^]^ as illustrated in Figure 3d. Remarkably, an evident alteration in the signal, surpassing the level of the LOI, is registered even when assaying only 12 ± 3 MUC1 molecules in the 0.1 mL sampled solution. Similar data were observed for CD55 and *KRAS*, and the capability of single‐molecule sensing was also demonstrated.^[^ ^35^ ^]^

#### 2.1.3 SiMoT Assay of 39 Patients’ Fluids

**Figure 4:**
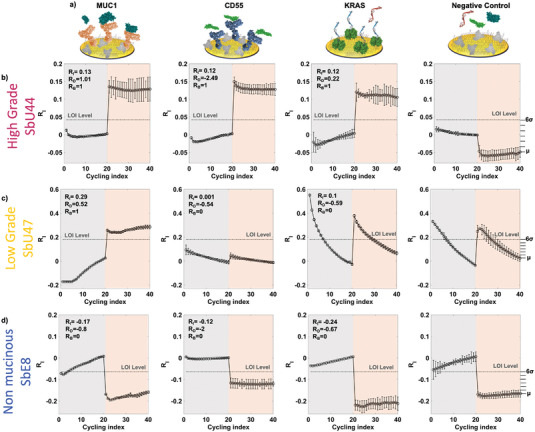
a) Pictorial representation of 3D sensing gate detecting interface featuring the chem‐SAM, the anti‐MUC1, and anti‐CD55 capturing antibodies and the b‐KRAS probe, along with the target markers. Also, the sketch of the gate coated with BSA serving as the negative‐control experiment is depicted on the right. In the other panels, the typical I_D_ current shifts R_I_ = (I – I^0^)/ I^0^ are plotted against the cycling index for b) a high‐grade mucinous cyst, c) a low‐grade mucinous cyst, and (d) a non‐mucinous cyst. R_I_ values are recorded at V_GS_ and V_D_ of −0.4 V, for each cycling index. The data points denote mean values derived from three replicates across three separate gates, with error bars calculated as relative standard deviations. The grey shaded area represents data collected post‐exposure to PBS reference fluid, while the red shading indicates measurements acquired following the exposure to the patient's specimen. The Limit‐of‐Identification (LOI) threshold is demarcated by a dashed‐dotted grey line.

The SiMoT array sensing protocol was engaged in the analysis of cyst fluid and blood plasma of 39 patients, as reported in **Table** **2**. The condition of each patient was evaluated through standard diagnostic methods involving the use of demographic information, histo/cytological analysis, and Next‐Generation Sequencing (NGS) of cell‐free DNA. For each patient, the oncoproteins MUC1 and CD55, the oncogene *KRAS* were assayed with the SiMoT assay in triplicate. Moreover, 7 negative‐control experiments were recorded for each specimen to evaluate the LOI level, as previously detailed. **Figure** **4**a displays the diagrams of the gate‐detecting interface, integrating the covalently bounded biorecognition layer (anti‐MUC1, anti‐CD55, b‐KRAS, and BSA) and their corresponding target analytes. Figure 4b displays the typical relative current changes (*R_I_ = (I – I^0^)/I^0^*) in relation to the cycling index preceding and subsequent to the exposure both to the reference fluid and the cyst fluid of a high‐grade mucinous cyst (patient SbU44, Table 2).

**Table 2:** List of the patients’ specimens, along with the state‐of‐the‐art diagnosis. Average values of the R_I_, R_D_ and R_B_ extracted for the SiMoT assay of MUC1, KRAS, and CD55, and of R_i_ and R_B_ extracted for the SIMOA assay of MUC1 and CD55 over three replicates.

			SiMoT									SIMOA			
			MUC1			CD55			KRAS			MUC1		CD55	
	Patient	Fluid	R_I_	R_D_	R_B_	R_I_	R_D_	R_B_	R_I_	R_D_	R_B_	R_i_	R_B_	R_i_	R_B_
High Grade	SbU25	Cyst	0.07	0.66	1	0.12	0.55	1	0.16	0.83	1	0.52	1	‐0.07	0
	SbU44	Cyst	0.13	1.01	1	0.12	0.22	1	0.11	‐2.49	1	0.02	0	‐0.19	0
	SbE32	Cyst	0.19	2.82	1	0.09	0.17	1	0	0	0	0.85	0	0.08	0
	SbE41,2	Cyst	0.17	0.59	1	0.11	0.23	1	0.01	0.25	0	0.55	0	‐0.05	0
Low Grade	SbE33b	Plasma	0.37	0.51	1	0.11	‐0.7	0	0.09	‐1	0	1.73	1	1.55	1
	SbE31.3b	Plasma	0.18	1.09	1	0.04	‐6.67	0	0.01	‐0.78	0	0.06	0	0.03	0
	SbE53	Plasma	0.08	0.63	1	‐0.29	‐0.64	0	‐0.26	‐1.39	0	0.66	1	12.35	1
	SbE3	Cyst	0.48	1.4	1	0.32	‐1.91	0	0.34	‐2	0	44.13	1	‐0.35	0
	SbE49b	Plasma	0.19	1.69	1	‐0.12	‐0.29	0	‐0.02	0.07	0	0.03	0	‐0.04	0
	SbU47	Cyst	0.29	0.52	1	0.001	‐0.54	0	0.1	‐0.59	0	0.50	0	‐0.25	0
	SbE37	Cyst	0.07	1.4	1	‐0.02	‐1.05	0	0.05	‐1	0	0.05	0	0.01	0
	SbE39	Cyst	0.21	1.02	1	‐0.22	0.69	0	‐0.21	0.53	0	8.68	1	2.89	1
	SbE55	Cyst	0.13	0.23	1	‐0.12	0.82	0	‐0.11	‐0.08	0	‐0.18	0	‐0.29	0
	SbE51	Cyst	0.34	1.64	1	0.27	‐2.26	0	0.22	‐0.7	0	1.29	1	0.41	0
Non‐mucinous	SbE50	Cyst	0.11	3.03	0	0.14	‐0.15	0	0.05	‐1.24	0	0.03	0	‐0.06	0
	SbE50b	Plasma	‐0.37	‐0.41	0	‐0.26	‐0.63	0	‐0.32	‐0.67	0	‐0.11	0	‐0.01	0
	SbE2	Cyst	0.14	1.31	0	0.05	‐0.46	0	0.04	‐0.07	0	0.26	0	0.21	0
	SbE4	Cyst	‐0.11	‐0.43	0	‐0.02	‐0.48	0	‐0.09	‐0.31	0	7.44	1	0.72	0
	SbG21	Cyst	‐0.31	‐1.26	0	‐0.28	‐3.77	0	‐0.31	‐0.65	0	0.98	1	0.61	0
	SbE19b	Plasma	0.16	0.95	0	‐0.3	‐0.57	0	‐0.3	‐0.69	0	‐0.09	0	‐0.02	0
	SbU22	Cyst	0.06	1.03	0	‐0.10	0.96	0	‐0.06	1.14	0	1.61	1	0.80	1
	SbU23	Cyst	0.22	0.91	0	0.09	3.03	0	0.09	0.36	0	0.90	1	0.35	0
	SbU27	Cyst	0.11	12.62	0	0.02	0.86	0	0.03	1.13	0	6.66	1	3.59	1
	SbU41	Cyst	0.16	1.06	0	0.11	13.4	0	0.15	2.42	0	0.27	0	‐0.10	0
	SbE8	Cyst	‐0.17	‐0.8	0	‐0.12	‐2	0	‐0.24	‐0.67	0	0.72	1	0.00	0
	SbE10	Cyst	‐0.04	‐0.92	0	‐0.07	‐0.25	0	‐0.04	‐0.6	0	3.37	1	‐0.47	0
	SbE22	Cyst	0.18	0.91	0	0	0	0	‐0.17	‐0.5	0	4.53	1	1.94	1
	SbE23	Cyst	‐0.02	‐2.26	0	0.09	‐0.26	0	0.23	‐1.11	0	0.60	0	0.14	0
	SbE24	Cyst	‐0.02	0	0	‐0.1	‐0.5	0	‐0.15	‐1.14	0	‐0.12	0	0.09	0
	SbE25	Cyst	0.12	1.11	0	0.03	1	0	0.01	0.14	0	‐0.04	0	‐0.04	0
	SbE29	Cyst	0	0	0	0	0	0	‐0.01	‐0.68	0	0.14	0	‐0.03	0
	SbE34	Cyst	0.15	0.52	0	0.21	1	0	0.19	0.67	0	0.03	0	‐0.07	0
	SbE35	Cyst	0.03	‐0.02	0	‐0.07	7.24	0	‐0.09	‐1.56	0	0.06	0	0.04	0
	SbE44	Cyst	‐0.90	1.01	0	‐0.06	1.39	0	‐0.07	0.61	0	0.17	0	‐0.25	0
	SbE45	Cyst	0.24	0.39	0	0.07	‐1.94	0	0.08	0.73	0	1.51	1	‐0.10	0
	SbE46	Cyst	0.18	0.75	0	0.07	‐0.66	0	0.14	2.73	0	0.47	0	‐0.37	0
	SbE47	Cyst	0.04	0.71	0	0.04	0.41	0	‐0.02	1.24	0	9.71	1	1.33	1
	SbE59	Cyst	0.17	0.63	0	0.06	2.80	0	0.06	0.11	0	0.98	0	‐0.15	0
	SbG12	Cyst	0.06	0.46	0	0.02	‐1.5	0	‐0.04	‐4	0	6.95	1	0.14	0

The data points depicted in Figure 4 are the mean derived from triplicates across three separate gates, and the error bars are calculated as the dispersion of the MUC1, CD55, and *KRAS* assays’ results from left to right. The panel on the far right illustrates the outcomes of the negative‐control experiments, alongside the previously defined LOI level. This level is indicated in the results of the MUC1, CD55, and *KRAS* assays, represented by a dashed‐dotted dark‐grey line. In Figure 4c,d, the relative current changes (*R_I_*) values are presented as a function of the cycling index, focusing on the analysis of cyst fluids from patients with low‐grade (SbU47) and non‐mucinous (SbE8) conditions. Those data allowed the extraction of three features, *R_I_*, *R_D_*, and *R_B_* defined as follows: i) *R_I_* = *(I_15‐20_ – I^0^_15‐20_)/I^0^_15‐20_* (average over three replicates), where $I_{15-20}=\frac{1}{5}\;\sum_{i\;=\;15}^{20}I\left(V_{GS},\;V_{D}=-0.4\;V\right)$ is the averaged sensing current derived from the last 5 cycles following the exposure to a patient specimen, while the $I_{15-20}^{0}=\frac{1}{5}\;\sum_{i\;=\;15}^{20}I^{0}\left(V_{GS},\;V_{D}=-0.4\;V\right)$ is the baseline current determined by averaging the readings from the last 5 cycles following the exposure in the PBS reference fluid. Therefore, *R_I_* represents the drain current change in the sensing signal relative to the baseline. *ii) R_D_* = $\frac{I_{20}-I_{1}}{I_{20}^{0}-I_{1}^{0}}$, where *I_1,_ I_20_, I^0^_1_, I^0^_20_* are the I_D_ values (at V_GS_ and V_D_ = −0.4 V) measured during the first and the last cycles. This characteristic corresponds to the normalized dynamic shift observed in a specific gate, considering the rate of change in current shift throughout the cycling process. *iii) R_B_* is a binary feature, being either 0 when the *R_I_* is lower than the LOI level or 1 when the *R_I_* is higher than the LOI level. Those features are presented in Table 2 for the samples from all assessed patients and serve as the defining elements for each sensing assay. These elements are utilized in the development of multivariate data processing through Principal Component Analysis (PCA), as detailed below (vide infra).

### 2.2 SIMOA SP‐X Assay of MUC1 and CD55 in Patients’ Fluid

The customized SIMOA SP‐X chemiluminescent assay was utilized to detect MUC1 and CD55 oncoproteins in the patients' cohort outlined in Table 2. This served as a benchmark for the SiMoT array analyses. Figure 1b,**c** illustrate the essential steps of the MUC1 and CD55 SIMOA assays, respectively. The SIMOA assay shares initial steps with conventional ELISA sandwiches. It employs a 96‐well ELISA plate with 12 spots, each having a diameter of 600 µm.^[^ ^17^ ^]^ In both cases, the immobilization of capture antibodies is accomplished via a peptide tag, connecting the chosen biorecognition elements to anchor antibodies that are densely printed on circular spots.^[^ ^50^ ^]^ The target analyte, whether MUC1 or CD55, is confined between the capture and the detector antibodies. In this investigation, analytes’ standard solutions, prepared in PBS reference fluid, were examined, covering concentrations ranging from 10 aM to 70 nM. The specific analyte is captured at the spot where either the anti‐MUC1 or anti‐CD55 capture antibodies are present. After exposure to analyte solutions, the process proceeds by exposing them to biotin‐labeled detector antibodies. Following this, the plate is subjected to washing to remove the antibodies in excess and is subsequently treated with streptavidin‐HRP to label the immunocomplexes with enzymes. Finally, luminol and H_2_O_2_ are introduced into each well. This enzyme‐substrate reaction generates locally emitted light from the immunocomplexes. The signal's strength is then correlated to the analyte concentration in the analyzed solution. The sensitivity of SIMOA assays for MUC1 and CD55 was enhanced using a design of experiments’ methodology with two variables, namely the concentrations of the capture and detector antibodies.^[^ ^35^ ^]^ According to prior findings, the assays were conducted, covering concentrations of 0.1 µg mL^−1^ for the capture antibodies, while 5 µg mL^−1^ of detector antibodies was used.^[^ ^50^ ^]^

**Figure 5:**
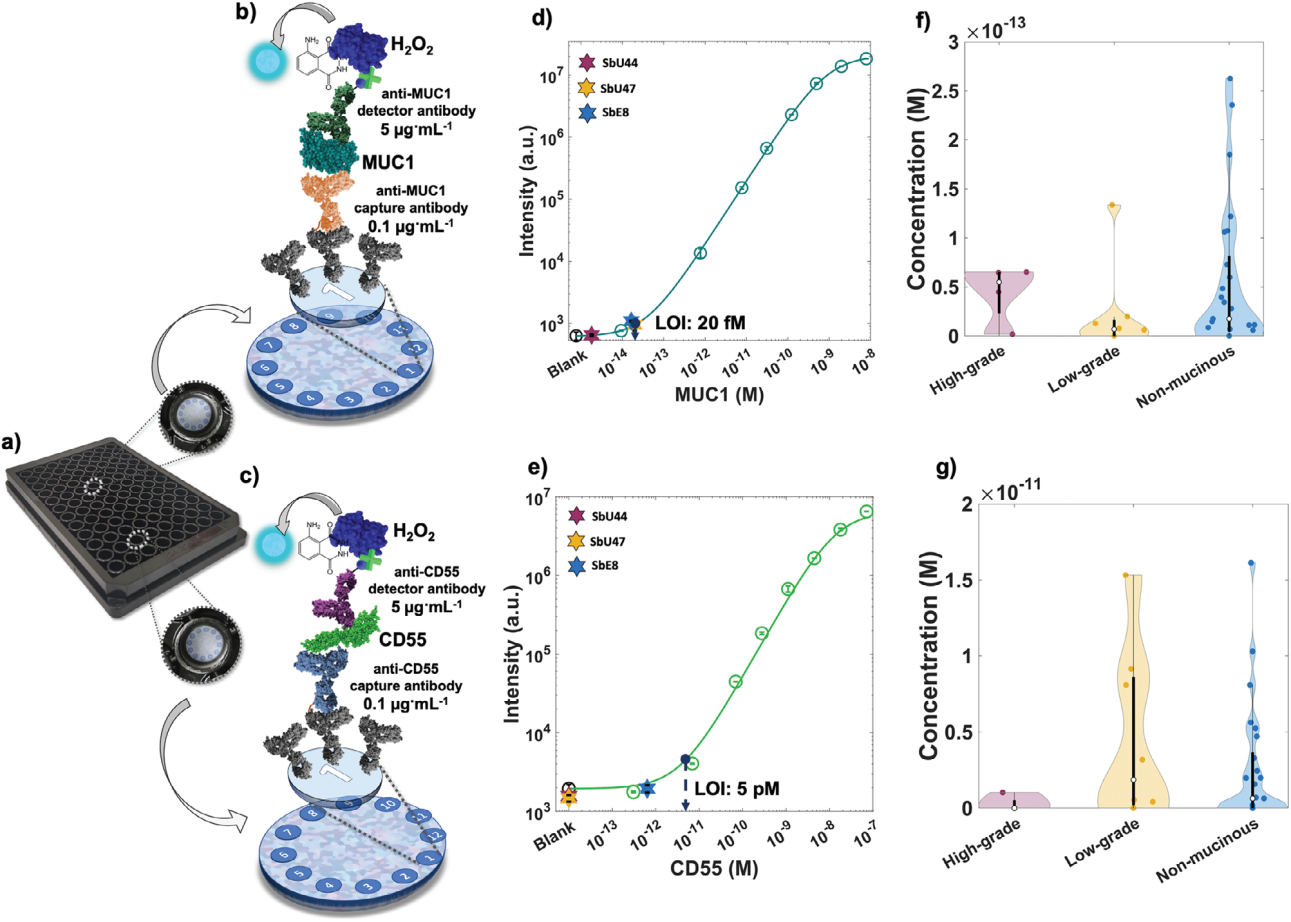
a) Image of the SIMOA SP‐X 96‐well ELISA plate. Workflow illustrating the SIMOA SP‐X assay designed for the detection of b) MUC1 and c) CD55 target analytes. Calibration curves for d) MUC1 and e) CD55 assays recorded with 0.1 µg mL^−1^ of capture antibodies (either anti‐MUC1 or anti‐CD55) and 5 µg mL^−1^of detector antibodies. MUC1 and CD55 standard solutions, prepared in PBS, were assayed, and presented as cyan and green hollow circles, respectively. In both cases, the blank is shown as black hollow circle. The sensing experiments were conducted in triplicates, while 6 blank replicates were acquired. The latter served to establish the Limit‐of‐Identification (LOI) achieved with the SIMOA assays. The modeling (solid curves) is performed using a logistic equation. The solid stars correspond to the SIMOA assay of cyst fluids from the high‐grade mucinous cyst SbU44, the low‐grade mucinous cyst SbU47, and the non‐mucinous cyst SbE8. Violin plots depict the distributions of concentrations for f) MUC1 and g) CD55 determined with the SIMOA assay for the 39 patients' samples.

**Figure** **5**d,e present the calibration curves, depicting the intensity of the chemiluminescent signal captured by the CCD camera and expressed in arbitrary units in relation to the concentration of the target analyte for the MUC1 and CD55 assays, respectively. The cyan and green hollow circles correspond to the average signal intensity observed upon exposure to standard solutions of MUC1 and CD55 analytes, respectively, evaluated in triplicates. Meanwhile, the black hollow circles represent the average signal from blank experiments, namely the signal registered upon exposure to the bare PBS reference fluid, conducted in six replicates.

The solid line represents the curve fit, employing a 5‐parameter logistic (5PL) equation, as follows:

(3) $$i=a+\frac{d}{{\left(1+{\left(\frac{x}{c}\right)}^{b}\right)}^{e}}$$

where *x* is either the MUC1 or CD55 concentration, while *i* is the chemiluminescent signal intensity. The parameters in Equation 3 are defined as follows: *a* corresponds to the initial response at *x* = 0, *d* represents the maximum signal intensity, *b* is the Hill Coefficient, *c* indicates the point of inflection in the dose‐curve, and *e* stands for the Skewness factor. The fitting process was iteratively performed, with multiple repetitions, adjusting the coefficients based on the residual errors observed in the preceding iteration. The blank experiments allowed to evaluate the LOI level of the SIMOA assays, as the average of the chemiluminescent signal intensity of the blank plus six times its standard deviation. Accordingly, a LOI of 20 fM was obtained for the MUC1 assay, while a LOI as high as 5 pM was obtained for CD55. The patients’ samples cohort, as detailed in Table 2, underwent analysis against MUC1 and CD55 using SIMOA assays in duplicate. Figure 5d,e display the average signal intensity for MUC1 and CD55 assays among high‐grade (SbU44, red star), potentially low‐grade (SbU47, orange star), and potentially non‐mucinous (SbE8) patients. Notably, all chemiluminescent signals from these samples fall below the LOI level. In Figure 5f,g, the distribution of MUC1 and CD55 concentrations, as registered with SIMOA technology, is depicted for all patients’ samples outlined in Table 2. It is evident from the presented violin plots that the variation in concentrations of both MUC1 and CD55 assayed with SIMOA among samples with high‐grade, potentially low‐grade, and potentially non‐mucinous cysts did not achieve statistical significance. As a further step, the two features *R_i_*, and *R_B_* were determined for each sample assayed with SIMOA. Those two features were defined as follows: *i) R_i_ = (i‐i^0^)/i^0^* where *i* and *i^0^* are the average chemiluminescent signal registered for the assayed sample and for the blank, while ii) *R_B_* as for SiMoT assay is a binary variable, defined either as 0 when the *R_i_* is lower than the LOI level or 1 when the *R_i_* is higher than the LOI level. Those features are presented in Table 2 for the samples from all assessed patients and were engaged in the development of the SIMOA assay's PCA model, as detailed below (vide infra).

### 2.3 Benchmarking SiMoT Array with SIMOA SP‐X Technology

Multivariate statistical analysis employing Principal Component Analysis (PCA) was conducted to assess and compare the SiMoT array multiplexing analysis with the SIMOA SP‐X assay. PCA, a statistical approach predominantly utilized for exploratory multivariate data analysis, identification of anomalies, and visualization of groups structures in the data, allows the derivation of an optimal number of principal components without losing information.^[^ ^51^ ^]^ PCA employs orthogonal transformations of a set of variables with interdependencies into a new set of variables that are linearly uncorrelated, commonly known as principal components (PCs). These new variables represent a standardized linear combination of the original variables, thereby fostering a comprehensive understanding of intricate multivariate phenomena. Moreover, PCA offers the ability to reduce the dimensions of the dataset by identifying the linear compositions of the original variables with the highest explained variance. This reduction in dimensionality contributes to a more streamlined and interpretable representation of the underlying multivariate relationships.

For this purpose, a PCA analysis was conducted involving the SiMoT assay, comprising 9 features for each patient pre‐treated with autoscaling.^[^ ^52^, ^53^ ^]^ Specifically, 3 features (R_I_, R_D_, and R_B_) corresponding to each of the 3 markers assessed (MUC1, CD55, and *KRAS*) were considered to develop the PCA model.

**Figure 6:**
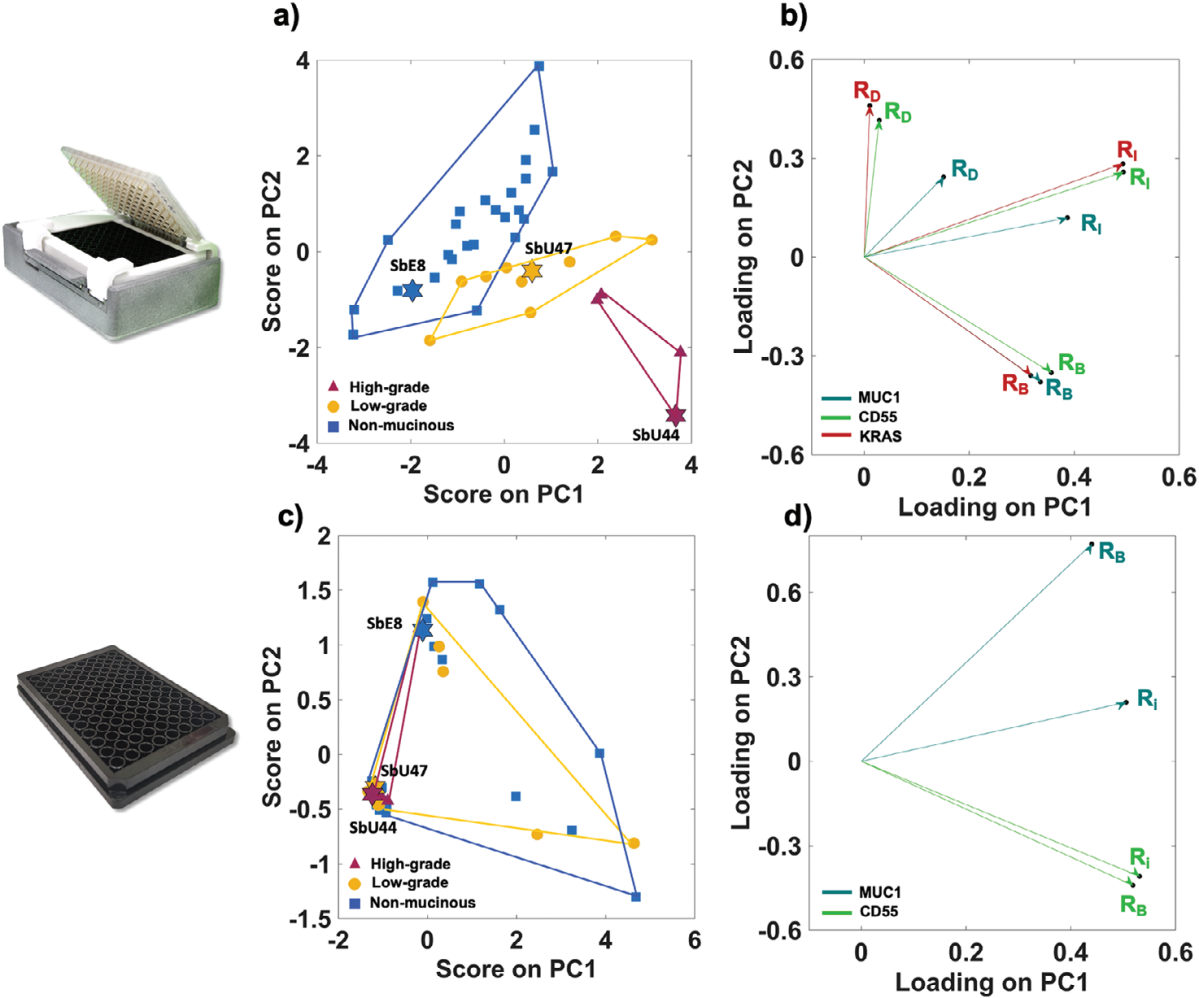
Multivariate data processing of the SiMoT and SIMOA assays a) Score‐Plot illustrating the scores of individual samples assessed using the SiMoT array for principal components PC1 and PC2. Red‐labelled samples correspond to high‐grade mucinous cysts, orange labels represent low‐grade mucinous cysts, and blue labels indicate non‐mucinous samples. b) Loading plot displaying the loadings of each initial variable extracted for the SiMoT assay on PC1 and PC2; R_I_, R_D_, and R_B_ are marked with cyan, green, and red arrows for MUC1, CD55, and KRAS, respectively. c) Score plot showing the scores of PC1 and PC2 obtained on the SIMOA assay on the 39 patients specimens. d) Loading plot illustrating the loadings of each original variable extracted for the SIMOA assay on PC1 and PC2; R_i_ and R_B_ are indicated with cyan and green arrows for MUC1 and CD55, respectively.

The first two principal components, PC1 and PC2, account for a substantial 51% of the variance, serving as the sole components required to characterize the entire dataset. In contrast, the higher‐order principal components primarily contribute to representing the noise level inherent in the assay. **Figure** **6**a depicts the *scores* on PC1 and PC2, portraying the transformed values of the original variables onto the new coordinate system defined by the principal components (PCs). The score plot in Figure 6a elucidates a distinct graphical clustering of high‐grade mucinous cysts, represented by red triangles. Notably, there is a discernible partial overlap among low‐grade, depicted as orange circles, and potentially non‐mucinous cysts samples, represented by blue squares. Specifically, the mucinous high‐grade cysts group in the area of the score plot featured by positive scores of PC1 and negative scores of PC2, while the potentially low‐grade samples cluster at negative scores of PC2. The potentially non‐mucinous samples group at negative scores of PC1. However, a confusion area can be observed for the potentially low‐grade and the potentially non‐mucinous cysts. The stars emphasized in the score plot represent the data points linked to the signal patterns acquired from the samples illustrated in Figure 4. Figure 6b illustrates the loadings on PC1 and PC2, offering insight into the contribution of each original variable to the formation of the principal components. The loading plot evidences that the presence of the three markers above the LOI decision threshold, namely R_B_ values of 1, leads to positive PC1 loadings and negative PC2 values. On the other hand, higher R_D_ values, accounting for the current dynamic behaviour upon cycling, lead to positive loading on PC2. Indeed, the dynamic processes occurring on the sensing gate surfaces are significantly influenced by the interaction between the biorecognition elements and their affinity ligands.^[^ ^32^ ^]^ It is thus possible to infer that the information present in the 9 variables obtained with the multiplexing SiMoT array is sufficient to clearly discriminate either the high or potentially low‐grade cysts, and the potentially benign specimens (non‐mucinous). While PCA served as a straightforward yet powerful method for extracting insights from complex systems, it is not ideally suited for predictive modelling. To address predictive modelling, one should turn to classification algorithms, recognized as supervised techniques designed for the specific task of predicting cyst grading, thus allowing to sort the confusion area observed in Figure 6a.^[^ ^35^ ^]^ However, these algorithms lie beyond the scope of this study, which is primarily focused on benchmarking the SiMoT technology against the SIMOA assay.

PCA multivariate data processing was undertaken also on the 4 autoscaled variables extracted from the SIMOA assay. Specifically, 2 features (R_i_, and R_B_) corresponding to each of the 2 markers assessed (MUC1, CD55) were considered to develop the PCA model. Even in this case the first two principal components, PC1 and PC2, were engaged in the characterization of the whole dataset, explaining the 88% of the variance. From the score plot in Figure 6c, it can be seen that the plane PC1‐PC2 is not capable of discriminating the three classes of pancreatic cyst's lesions. Indeed, the scores of PC1 and PC2 exhibit a random distribution around the center of the score plot. Additionally, the loading plot in Figure 6d illustrates that the detection of either one of the two markers is indicated by positive PC1 loadings. However, it is possible to partially decouple the two protein markers, resulting in reverse PC2 loadings. Specifically, CD55 is primarily described by negative PC2 loadings (green arrows in Figure 6d), while MUC1 by positive loadings on PC2 (green arrows in Figure 6d). The data presented in Figure 6c,d collectively imply that the sensitivity of the SIMOA assay is inadequate for the accurate identification of high‐grade mucinous cysts. These samples exhibit consistent negative loadings on PC1, signifying that the SIMOA assay failed to detect both MUC1 and CD55. Furthermore, samples classified under the potentially non‐mucinous cysts category demonstrate positive scores on PC1, indicating a potential lack of specificity in the SIMOA assay.

Evidently, the SiMoT array surpasses SIMOA technology in terms of accuracy, particularly with regard to diagnostic sensitivity. Furthermore, SIMOA technology allows for the analysis of protein markers whereas SiMoT technology facilitates the testing of both protein markers and gene mutations (akin to NGS) using a singular device. Additionally, the SiMoT array guarantees label‐free detection for a single marker, streamlining the sample preparation to the bare minimum. Notably, the SiMoT array delivers results for at least 5 patients within just 1.5 hours, while SIMOA requires approximately 5 hours, at roughly comparable costs. Furthermore, the electronic reader and data collection system enable a simple and convenient remote control of the entire assay procedure. Comprehensive data connectivity supports integration with digital platforms and guarantees smooth access to electronic health records, thereby enhancing the efficiency of data management, analysis, and reporting.

## 3 Conclusion

To conclude, the assessment of the SiMoT array technology for the early detection of precursor cysts related to pancreatic cancer is conducted, benchmarking it against the commercially available SIMOA SP‐X Planar assay developed by Quanterix. The SiMoT array integrates a one‐use cartridge with a form factor resembling that of an 8×12 ELISA plate, complemented by a lid of 3D‐printed sensing gates extending into each well. Furthermore, it features a reusable reader, inclusive of the PCB module and the Si‐IC, which connects to a standard smart device via USB for operation. The SiMoT array demonstrates a distinctive capability by reliably conducting concurrent assays for single oncoproteins and single‐copy oncogenes. Within this system, oncoproteins MUC1 and CD55, along with the oncogene *KRAS*, are identified in either cyst fluid or blood plasma specimens from a cohort of 39 patients. The objective of this analysis is to distinguish mucinous cysts and pinpoint those exhibiting high‐grade characteristics, acknowledged as early indicators of pancreatic malignancy. The simultaneous identification of the three target biomarkers is carried out in triplicate, with seven experiments serving as negative controls. The testing encompasses 5 to 6 distinct patients for each plate. The same cohort of patients underwent analysis using the commercially available SIMOA SP‐X technology to identify oncoproteins MUC1 and CD55. A multivariate statistical analysis, utilizing principal component analysis (PCA), was devised and fed with the data obtained from both technologies. The PCA analysis unveiled that the SiMoT array, with its multiplexing capabilities, distinctly discriminates high‐grade, low‐grade, and non‐mucinous cysts. In contrast, the PCA conducted on SIMOA assay analysis showed no significant differences among the three classes. Furthermore, the SiMoT technology stands out as a faster alternative compared to the SIMOA SP‐X assay. Notably, SiMoT is the sole assay that enables the concurrent examination of oncoprotein and oncogenes markers with a Limit‐of‐Identification of one single entity. The SiMoT technology additionally furnishes an electronic response, making it well‐suited for direct digital data communication. This capability opens avenues for the development of field‐deployable liquid‐biopsy diagnostics.

## 4 Experimental Section

### Materials

High‐purity ethanol, P3HT, and PBS tablets, BSA and avidin were all obtained from Merck without additional purification steps. Poly(ethylene 2,6‐naphthalate) (PEN) substrates (125 µm) were sourced from Du Pont. Monoclonal antibodies for Mucin 1 and the recombinant protein of human MUC1 were supplied by OriGene, while CD‐55 monoclonal antibodies and the human CD55 proteins were contributed by Abnova. Thermo Fisher Scientific provided mutated nucleic acids KRAS (KRAS^G12D^) along with the Biotinylated‐KRAS^G12D^ fwd probe. MilliporeSigma contributed self‐assembled monolayers. Quanterix supplied all the reagents for the development of the SIMOA assays and provided pre‐spotted ELISA plates with anchor antibodies, stored at 2−8 °C.

### EGOFETs Array Fabrication

Employing a flexible PEN foil, a lift‐off photolithography technique was applied to design gold electrodes on the PEN substrate. A 2 nm chromium adhesion layer preceded the evaporation of 30 nm gold. Subsequent to standard cleaning procedures in solvents at increasing polarity, and a 2‐minute oxygen plasma treatment, an interdigitated source and drain electrode structure was selected to enhance transistor's electronic performance levels. The configuration featured a 10^4^ µm channel width, 5 µm channel length, and a coplanar gate with a 2.5 mm diameter. A SU8 inkjet‐printed film was deposited onto the gold connectors, thus excluding the electronic channel area and coplanar gate. Subsequently, the organic semiconductor P3HT was selectively printed on gold source and drain contacts.

### 3D Cover Plate Fabrication

The SiMoT sensing gate cover plate was fabricated using 3D printing stereolithography, the samples underwent post‐curing through annealing and UV exposure. Samples were heated in a UV stove (20 min., 65°C) and further exposed to UV light (30 min.). To reduce 3D sensing gate plate roughness prior to electron‐beam evaporation, a 2 µm Parylene‐C layer was grown using a CVD (chemical vapor deposition) approach. Subsequently, a 150 nm thick gold layer was e‐beam evaporated onto the planarized sensing gate plate. Afterwards standard cleaning procedures with solvent at increasing polarity and ozone cleaning were accomplished, prior to the biofunctionalization.

### 3D Sensing Gates Biofunctionalization

Biofunctionalization of the gate begins by immobilizing a chemical self‐assembled monolayer on the detecting interface. To this aim, a solution comprising 10 mM of 3‐mercaptopropionic acid (3‐MPA) and 11‐mercaptoundecanoic acid (11‐MUA) in ethanol, with a molar ratio of 10:1 were used. Then the gates were exposed to 1‐Ethyl‐3‐(3‐dimethylaminopropyl) carbodiimide (EDC, 200 mM) and sulfo‐N‐hydroxysuccinimide (sulfo‐NHS, 50 mM) solution in water (20 min., 21 °C). Subsequently, the biofunctionalization protocol advances as follows. i) The gate detecting interface was immersed in PBS solutions either of anti‐MuC1 or anti‐CD55 (2 hours, 21°C), and then to ethanolamine 1 M in PBS (45 min., 21 °C). Finally, the bio‐functionalized gate was immersed in a BSA solution in PBS (1 hour, 21 °C). ii) The gate pillar was submerged in an AV solution in PBS (2 h, 21 °C). Following this, the AV‐modified SAM undergoes further treatment with ethanolamine 1 M in PBS (45 min., 21 °C). Afterward, the gate was immersed in a 0.5 µM b‐KRAS PBS solution (1 h, 21 °C). In the negative control experiments, only BSA was covalently bounded to the chem‐SAM.

### Collection of Body Fluids

All specimens were obtained during routine hospitalization procedures, processed at the Düsseldorf Institute of Pathology, and subsequently stored at −80 °C. Pancreatic cyst juices (1‐5 mL) were acquired through Fine‐needle biopsy under endoscopic ultrasound guidance. Venous blood was gathered in BCTs tubes, adhering to the manufacturer's instructions. To eliminate any residual cells, pancreatic cyst fluids underwent centrifugation (1600 × g, 10 min., 21 °C). The resulting human cyst fluids were then received in a diluted form at a ratio of 1:8 (v/v) in PBS diluent. Human blood plasma samples were subjected to centrifugation (10000 × g, 5 min., 21 °C). Subsequently, these plasma samples were diluted at a ratio of 1:8 (v/v) with PBS diluent afore undergoing analysis. In preparation for analysis with b‐KRAS modified gates, a portion of each diluted sample, whether from plasma or cyst fluids, was heated in hot bath (3 min., 90 °C).

### SiMoT Array Sensing Protocol

The 8 × 12 SiMoT array was fully immersed in HPLC‐grade water for ≈24 h. To stabilize the source‐drain current (ID), a cyclic measurement of transfer characteristics was counted using the reference lateral gate. This process entailed recording I_D_ versus V_GS_ at a constant V_D_ every thirty minutes until the current drift diminished to below 5% per day. As a preliminary step, the sensing gate plate was exposed to PBS (serving as a reference fluid) (0.1 mL in each well, for 10 minutes). Following this, the plate underwent a thorough wash with HPLC water, and a stable baseline (*I^0^*) was established by measuring a *cycling* over 2.5 min. Next, the same gate plate was exposed to the patients’ specimen diluted at a ratio of 1:8 (v/v) in PBS (10 min.). After thorough washing with HPLC water, a second cycling was acquired. Throughout both incubation phases, the SiMoT array electrical reliability was monitored by recording the I_D_ traces with the gold LG defined at the bottom of each well. The analysis of all collected data was performed using the MatLab software.

### SIMOA SP‐X Planar Oncoproteins´ Assays

Following established procedures, anti‐MUC1 and anti‐CD55 detection antibodies underwent PBS exchange and subsequent biotinylation using NHS‐PEG4‐Biotin. Excess biotin removal was achieved by dialysis into PBS using an Amicon filter. Calibrator antigen stocks for MUC1 and CD55 were prepared through serial dilution in PBS. Custom ultrasensitive assays were developed for both MUC1 and CD55. In accordance with the developed protocols, anchor antibodies were pre‐spotted on microplates and washed with Tris buffer and Tween 20 cleaner. Each microplate was incubated with the peptide‐tagged anti‐MUC1 or anti‐CD55 capture antibodies' solution at a concentration of 0.1 µg mL^−1^ (30 min., 21 °C) with agitation (525 r.p.m.) on an orbital microplate shaker. Subsequently, the microplate underwent washing, and MUC1 or CD55 standard solutions, with concentrations ranging from 10 aM to 70 nM were incubated for 120‐minute. Then, the microplate was washed and incubated with biotinylated anti‐MUC1 or anti‐CD55 detection antibodies for 30 minutes. Following this, streptavidin‐horseradish peroxidase was added to each well (30 min., 21 °C). Finally, luminol and hydrogen peroxide were added to each well. The SIMOA SP‐X (Quanterix Corp.) was employed for imaging the microplate. Specifically, the chemiluminescent signal emitted through the transparent bottoms of all the wells of the microtiter plate was collected by a lens and imaged onto a cooled 3.2 Mp CCD camera. The resulting image had a resolution of ≈46 µm per pixel. These custom assay conditions underwent optimization through a replicated, two‐factors (namely the concentrations of the capture and detector antibodies), full factorial design. The limit of identification (LOI) served as the response in the factorial design.^[^ ^54^ ^]^ The experimental design's optimized settings were applied to develop the assay for measuring MUC1 and CD55 in patients specimens. Each sample was assayed in duplicate, and the signal intensity was captured by the CCD camera.

## Conflict of Interest

The authors declare no conflict of interest.

## Data Availability

The data that support the findings of this study are available from the corresponding author upon reasonable request.
